# Integrative single-cell and bulk transcriptomic profiling identify peripheral T-cell-associated candidate biomarkers in community-acquired pneumonia

**DOI:** 10.7150/ijms.136006

**Published:** 2026-08-11

**Authors:** Huachi Jiang, Xiaodong Fan, Shufang Zhou, Jia Hua, Chu Qin, Haoda Yu

**Affiliations:** 1 Department of Respiratory Medicine, The Affiliated Wuxi People's Hospital of Nanjing Medical University, Wuxi 214023, China.; 2 Department of Thoracic Surgery, The Affiliated Wuxi People's Hospital of Nanjing Medical University, Wuxi 214023, China.; 3 Department of Nephrology, The Affiliated Wuxi People's Hospital of Nanjing Medical University, Wuxi 214023, China.

**Keywords:** community-acquired pneumonia, peripheral blood mononuclear cells, single-cell RNA sequencing, transcriptomics, biomarkers

## Abstract

**Background:**

Community-acquired pneumonia (CAP) is associated with marked immune dysregulation, but the peripheral T-cell transcriptional alterations linked to CAP remain incompletely defined. We aimed to characterize CAP-associated T-cell heterogeneity and identify candidate blood biomarkers by integrating single-cell and bulk transcriptomic data.

**Methods:**

GEO-processed whole-blood microarray expression data from 108 patients with CAP and 42 healthy controls (GSE65682), together with processed PBMC scRNA-seq data from five non-influenza CAP donors and four control donors (GSE164948), were analysed. Cell annotation, differential expression, disease contribution scoring, LASSO regression, and support vector machine-recursive feature elimination were used to identify candidate genes. Immune infiltration, enrichment, and pseudotime analyses were performed. Key findings were evaluated by qRT-PCR in PBMCs from an independent cohort of 36 CAP patients and 18 healthy controls.

**Results:**

T cells showed the highest disease contribution score and were enriched for pathways related to antigen processing, phosphorylation, and immune regulation. Four candidate biomarkers, CD8B, GZMM, MYL12B, and TUBA1A, showed differential expression between CAP and controls. CIBERSORT suggested increased eosinophils and monocytes and reduced resting CD4+ memory T cells in CAP. Pseudotime analysis indicated that CAP-associated T cells accumulated at higher inferred pseudotime values. qRT-PCR confirmed significant upregulation of these four candidate biomarkers in CAP PBMCs; FCGR2A also showed CAP-associated expression changes.

**Conclusions:**

Integrative transcriptomic profiling identified peripheral T-cell dysregulation in CAP and highlighted CD8B, GZMM, MYL12B, and TUBA1A as candidate blood biomarkers. These findings provide a basis for further multicenter validation and mechanistic studies.

## Background

Community-acquired pneumonia (CAP) is defined as acute inflammation of the lung parenchyma occurring outside a hospital or healthcare facility. The pathogenesis of CAP involves rapid bacterial, fungal, or viral replication within alveoli and small airways, triggering host inflammation that disrupts pulmonary homeostasis. This leads to local symptoms (dyspnea, cough, impaired gas exchange, and radiographic consolidation) and systemic manifestations (fever, fatigue, altered mental status, and potential sepsis) 1**.** As a critical illness of global public health concern, CAP continues to be the leading cause of death among infectious diseases worldwide, emphasizing the need for more effective prevention and management strategies 2. However, systematic characterization of peripheral T-cell heterogeneity at single-cell resolution and machine learning-selected candidate blood biomarkers for CAP remain largely lacking. Most studies focus on innate immunity or bulk transcriptomics alone, without integrating scRNA-seq, bulk RNA-seq, and clinical qRT-PCR validation.

CAP pathogenesis is increasingly recognized as a dynamic interplay between microbial virulence factors and host immune responses 3. The innate immune system initiates early defense through pathogen phagocytosis and inflammatory signaling, while T-cell-mediated responses shape pathogen clearance, inflammatory control, and tissue repair 4,5. However, most available studies emphasize systemic inflammatory markers or pathogen-specific responses, and the peripheral cellular programs associated with CAP remain incompletely characterized 6,7. CAP lacks rapid, objective, blood-based diagnostic biomarkers to assess immune status and guide early intervention. Peripheral T cells are easily accessible and closely reflect systemic immune dysregulation during CAP, making them ideal for biomarker discovery.

In recent years, bioinformatics approaches integrating single-cell and bulk transcriptomic data have been increasingly used to study the molecular mechanisms of pulmonary diseases. Single-cell analysis can resolve cellular heterogeneity and identify disease-associated cell states, thereby supporting biomarker discovery and therapeutic target prioritization 8. Lu et al. reported that tissue-resident MAIT17 cells are activated in infected respiratory mucosa and contribute to IL-17-mediated inflammation during CAP 9. Nevertheless, integrative analyses specifically focused on peripheral T-cell-associated candidate biomarkers in CAP remain limited.

To fill these gaps, we integrated single-cell and bulk transcriptomic data, used machine learning to select candidate genes, and evaluated candidate biomarkers in an independent clinical cohort by qRT-PCR. This study aims to reveal T-cell dysfunction and identify candidate peripheral blood biomarkers for CAP.

## Methods

### Data acquisition

Public transcriptomic datasets were obtained from the NCBI Gene Expression Omnibus (GEO). The GEO-provided processed whole-blood microarray series matrix for GSE65682 24 (Affymetrix Human Genome U219 Array, platform GPL13667), comprising 108 CAP samples and 42 healthy controls, was used. According to the GEO record, the raw CEL files had undergone RMA background correction, quantile normalization, median-polish summarization, variance-based probe filtering, and correction of non-experimental chip effects using surrogate variable analysis and ComBat. The deposited expression values were already RMA-normalized and log2-transformed. For the CAP-control comparison, we used the GEO-provided processed count matrix and cell metadata from GSE164948 25 (platform GPL20301), selecting five non-influenza CAP (CAP-other) samples and four healthy controls; the three CAP-Flu samples in the same file were excluded. The selected dataset contained 6,180 cells (3,987 CAP-other and 2,193 control cells) before secondary quality control.

### Single-cell data quality control

The GEO-provided processed count matrix and cell metadata were imported into Seurat. Quality-control metrics were recalculated from the deposited matrix for the secondary analysis. We inspected total UMI counts (nCount_RNA), detected genes (nFeature_RNA), and the percentage of mitochondrial transcripts (percent.mt) for each cell. Cells with low complexity or disproportionately high mitochondrial transcript percentages were considered low quality. Outliers were identified relative to the sample distributions using a three-MAD threshold, together with nFeature_RNA > 200, as specified below.

### Single-cell data dimensionality reduction clustering and cell annotation

We used Seurat's NormalizeData function (normalization.method = "LogNormalize") to normalize the data by dividing the feature counts in each cell by the total counts for that cell and multiplying by a scale factor of 10000, and then the data were log-transformed. The cell cycle score was calculated using CellCycleScoring. FindVariable-Features was used to identify highly variable genes. We used ScaleData to regress variation associated with mitochondrial and ribosomal transcript percentages and cell-cycle scores. The expression matrix was reduced linearly by RunPCA, and principal components were selected for subsequent analysis. The batch effect was removed via Harmony, and nonlinear dimensionality reduction was performed with RunUMAP (uniform manifold approximation and projection, UMAP). Cell identities were assigned using canonical markers (T cells: CD3D, CD3E, CD3G, and CD8B; NK cells: NKG7 and GNLY; B cells: MS4A1 and CD79A; monocytes: LST1, LYZ, S100A8, and S100A9; platelets: PPBP and PF4) and were cross-checked against the GEO-provided cluster metadata, CellMarker and PanglaoDB, the literature, and SingleR annotations.

Differential expression between CAP and control T cells was assessed at the individual-cell level using Seurat FindMarkers with the default Wilcoxon rank-sum test. Genes with an absolute average log2 fold change greater than 0.25 and a Bonferroni-adjusted P value less than 0.05 were retained. Donor identity was not included as a blocking variable or covariate, and neither pseudobulk aggregation nor a mixed-effects model was used. This analysis was therefore considered exploratory and hypothesis-generating.

### Determination of the contribution of cell subsets to disease

We characterize the contribution of different cell subpopulations to disease by considering changes in cell numbers and gene expression. First, we identified the characteristic genes of each subpopulation. In brief, we performed a batch differential gene expression analysis and described the changes in the differential genes from the disease group to the control group. We then defined the FCscore, which measures changes in the number and expression levels of characteristic genes during biological processes.

FCscore(i,j) = √[FCexp(i,j) × FCprop(j)] (1)

where FCexp(i,j) represents the expression fold change of characteristic gene i in cluster j, and FCprop(j) represents the fold change in the proportion of cells in cluster j. FCscore(i,j) is defined as the square root of the product of FCexp(i,j) and FCprop(j). Finally, the contribution of different cell subpopulations to disease was characterized by the average FCscore of all characteristic genes in the cluster.

### Functional analysis

The R package "ClusterProfiler" was used for functional annotation of genes to explore the functional correlation of marker genes in key cells comprehensively. Gene Ontology (GO) and Kyoto Encyclopedia of Genes and Genomes (KEGG) analyses were used to assess the relevant functional categories. Enriched GO terms and KEGG pathways with p values less than 0.05 were considered statistically significant.

### Feature selection process of the LASSO regression and SVM algorithms

First, marker genes in key cells were selected as candidate genes (|log2FC| > 0.25 and adjusted P < 0.05). We subsequently used LASSO regression and SVM-RFE to further screen disease-associated genes. The analysis used the GEO-provided processed GSE65682 microarray expression matrix. No additional log2 transformation or between-array normalization was performed. Probe sets were mapped to gene symbols, and expression values from multiple probe sets mapping to the same gene were averaged. Predictor standardization was performed internally using the default settings of glmnet and within the SVM-RFE procedure. CAP status was encoded as 1 and control status as 0. No separate held-out test set was used; model selection was based on 10-fold cross-validation with a fixed random seed to reduce overfitting. For LASSO regression, cv.glmnet selected lambda.min under 10-fold cross-validation, with L1 regularization used for feature selection. For SVM-RFE, recursive feature elimination was performed within each cross-validation fold, and features with the highest selection frequencies across folds were retained. The intersection of features selected by LASSO and SVM-RFE was defined as the candidate biomarker set.

### Immune infiltration analysis

The CIBERSORT method is a widely used method to evaluate immune cell types in the microenvironment. On the basis of the principle of support vector regression, this tool analyzes the expression matrix of immune cell subtypes via deconvolution. CIBERSORT contains 547 biomarkers that distinguish 22 human immune cell phenotypes, including T cells, B cells, plasma cells, and myeloid cell subsets and was used to analyze patient data in this study, and correlation analysis were performed on the gene expression and immune cell data.

### GSEA

The samples were divided into high-low-expression groups according to their expression levels of candidate biomarkers, and the differences in signaling pathways between the two groups were further analyzed by GSEA. Background gene sets were annotated gene sets of version 7.0 downloaded from MsigDB as annotation gene sets of subtype pathways. Differential expression analysis of pathways between different groups was performed. Significantly enriched gene sets (adjusted p value less than 0.05) were sorted according to consistency scores. GSEA is often used to explore the biological significance of genes and their roles in disease.

### GSVA

GSVA is a nonparametric, unsupervised method for evaluating transcriptome gene set enrichment. By comprehensively scoring the gene set of interest, GSVA converts the gene level change into a pathway level change and then judges the biological function of the sample. In this study, gene sets were downloaded from MsigDB, and the GSVA algorithm was used to obtain comprehensive scores for each gene set to evaluate potential biological functional changes in different samples.

### Pseudotime analysis

Single-cell technology has made it possible to elucidate the transcriptional regulation of complex physiological processes and highly heterogeneous cell populations. This technology enables the identification of genes that are characteristic of specific cell subtypes, genes that mark intermediate states of differentiation, and genes that differentiate between two different cell fates. In many single-cell studies, individual cells express genes in an unsynchronized manner, and each cell is in a specific state along their fate trajectory. Monocle v2.28.0 was used for pseudotime analysis. The normalized Seurat RNA expression matrix (GetAssayData, slot = "data") was converted to a CellDataSet; size factors and dispersions were estimated using estimateSizeFactors and estimateDispersions. Ordering genes were selected from dispersionTable using mean_expression >= 0.1 and dispersion_empirical >= dispersion_fit, applied with setOrderingFilter, and used for two-dimensional DDRTree reduction. Cells were ordered with orderCells, with the root state inferred by the default orderCells procedure. Because the data were cross-sectional, pseudotime was interpreted as an inferred transcriptional ordering rather than direct evidence of temporal differentiation or disease progression.

### Clinical Participants and Sample Collection

To evaluate PBMC-level expression of the identified candidate biomarkers, a clinical validation cohort was established. A total of 36 patients diagnosed with Community-Acquired Pneumonia (CAP) and 18 healthy volunteers were recruited from the Department of Respiratory and Critical Care Medicine at Nanjing Medical University affiliated Wuxi People's Hospital between May 2025 and Aug 2025.

The diagnosis of CAP was based on the guidelines of the American Thoracic Society/Infectious Diseases Society of America (ATS/IDSA). The exclusion criteria included: (1) Hospital-acquired pneumonia or ventilator-associated pneumonia; (2) Immunocompromised status; (3) Active malignancy; or (4) Pregnancy. The healthy control group consisted of individuals with no history of acute infection or chronic inflammatory diseases in the past three months. Demographic characteristics of the independent qRT-PCR validation cohort are summarized in Table 1. Participants were not individually matched; CAP participants were older than healthy controls (P < 0.001), whereas sex distributions were comparable (P = 0.776). Only age and sex were available for this cohort.

### PBMC Isolation and RNA Extraction

Peripheral blood samples (5 mL) were collected from all participants in EDTA-coated tubes. Peripheral blood mononuclear cells (PBMCs) were isolated within 4 hours of collection. Total RNA was extracted from isolated PBMCs using TRIzol reagent (Tiangen, China). RNA concentration and purity were assessed using a NanoDrop 2000 spectrophotometer (Thermo Fisher Scientific, USA), and samples with an A260/A280 ratio of 1.8-2.0 were used for downstream experiments.

### Quantitative Real-Time PCR (qRT-PCR)

Complementary DNA (cDNA) was synthesized from 1 μg of total RNA using the PrimeScript RT Reagent Kit (Takara, Japan). Because total RNA was obtained from unsorted PBMCs, the qRT-PCR results reflect PBMC-level expression and do not establish a T-cell-specific cellular source. qRT-PCR was performed on a Roche LightCycler 480 system using TB Green Premix Ex Taq II to quantify five selected genes (CD8B, FCGR2A, GZMM, MYL12B, and TUBA1A). The reaction conditions were 95°C for 30 s, followed by 40 cycles of 95°C for 5 s and 60°C for 30 s. Relative expression levels were calculated using the 2^-ΔΔCt method, with GAPDH as the endogenous control. Primer sequences are listed in Supplementary Table S1.

### Ethics Committee Approval and Patient Consent

This study was conducted in accordance with the Declaration of Helsinki and was approved by the Ethics Committee of Nanjing Medical University affiliated Wuxi People's Hospital (Approval No.KY25061). Written informed consent was obtained from all participants or their legal guardians.

## Statistical analysis

All statistical analyses were performed using R software (version 4.3.0) and GraphPad Prism (version 10.6.0). For bioinformatics data, differences between groups were analyzed as described in the respective sections.

For the clinical PCR validation data, the normality of the data distribution was assessed using the Shapiro-Wilk test. Comparisons between the CAP group and the control group were performed using a two-sided Welch's t-test, consistent with the prespecified Prism analyses. Correlation analysis between genes was assessed using Pearson's correlation coefficient. A P-value < 0.05 was considered statistically significant (*P < 0.05, **P < 0.01, ***P < 0.001, ****P < 0.0001). Age was summarized as median (interquartile range) and compared using a two-sided Mann-Whitney U test; sex was summarized as n (%) and compared using Fisher's exact test.

## Results

### Single-cell data quality control and cell annotation

Considering the data quality of multiple samples, cells with fewer than 200 captured genes were filtered out. The filtering criteria were nFeature_RNA > 200 and nFeature_RNA, nCount_RNA, and percent.mt no more than three MADs above their respective medians, where nFeature_RNA represents the number of genes, nCount_RNA represents the total UMI of the cell, and percent.mt represents the percentage of mitochondrial reads. The DoubletFinder package was then used to filter out the doublet cells, retaining 5,584 of the 6,180 selected cells, and a violin plot and scatter plot were generated (Figure S1A-B).

### Dimension reduction, clustering and cell annotation of single-cell data

**Figure 1:**
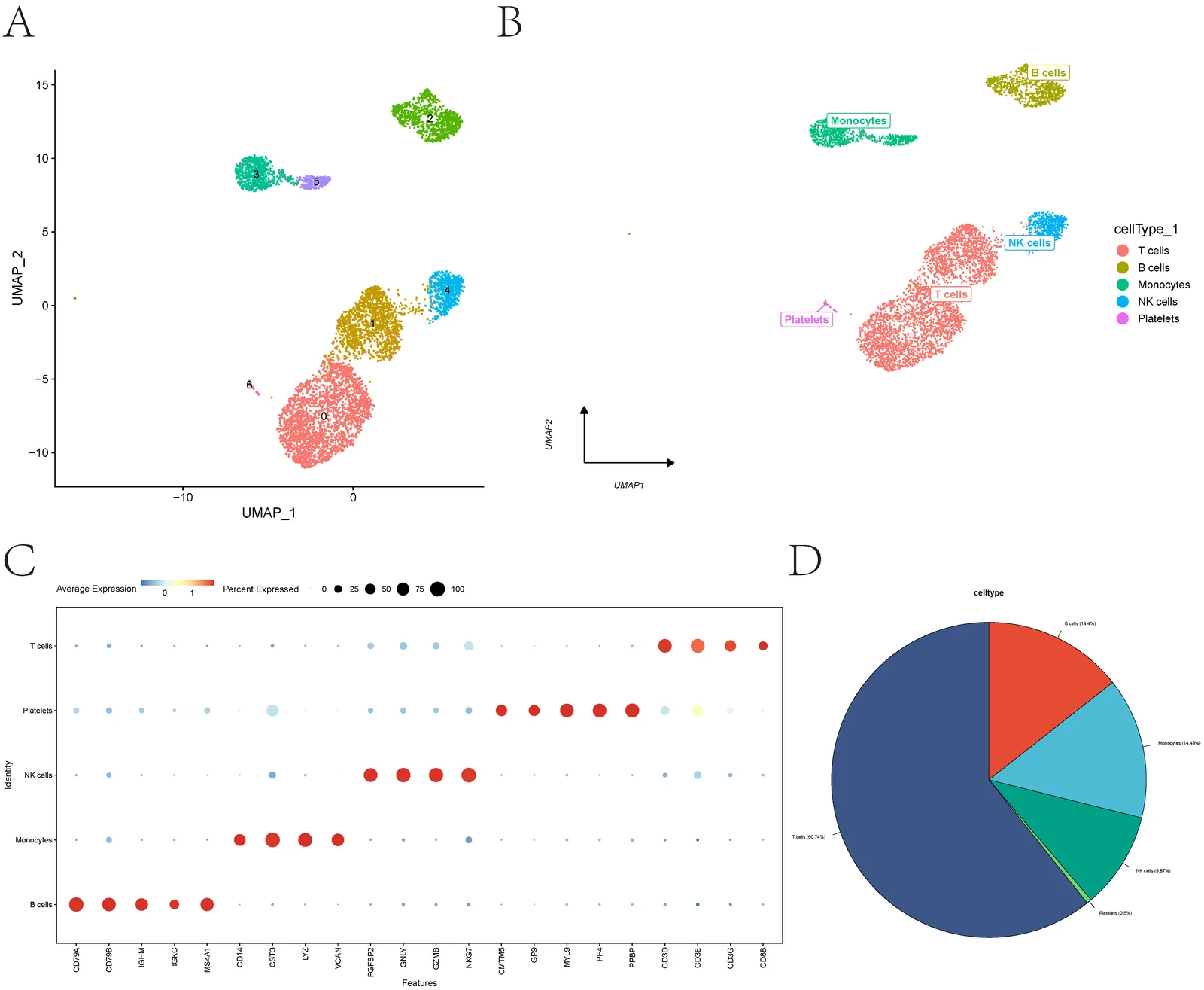
** Cell annotation. (A)** UMAP visualization of seven cell clusters derived from principal-component analysis. **(B)** Annotation of the seven clusters into five cell types: T cells, NK cells, B cells, monocytes, and platelets. **(C)** Dot plot of canonical markers used to annotate the five cell types. **(D)** Relative proportions of the five cell types.

**Figure 2:**
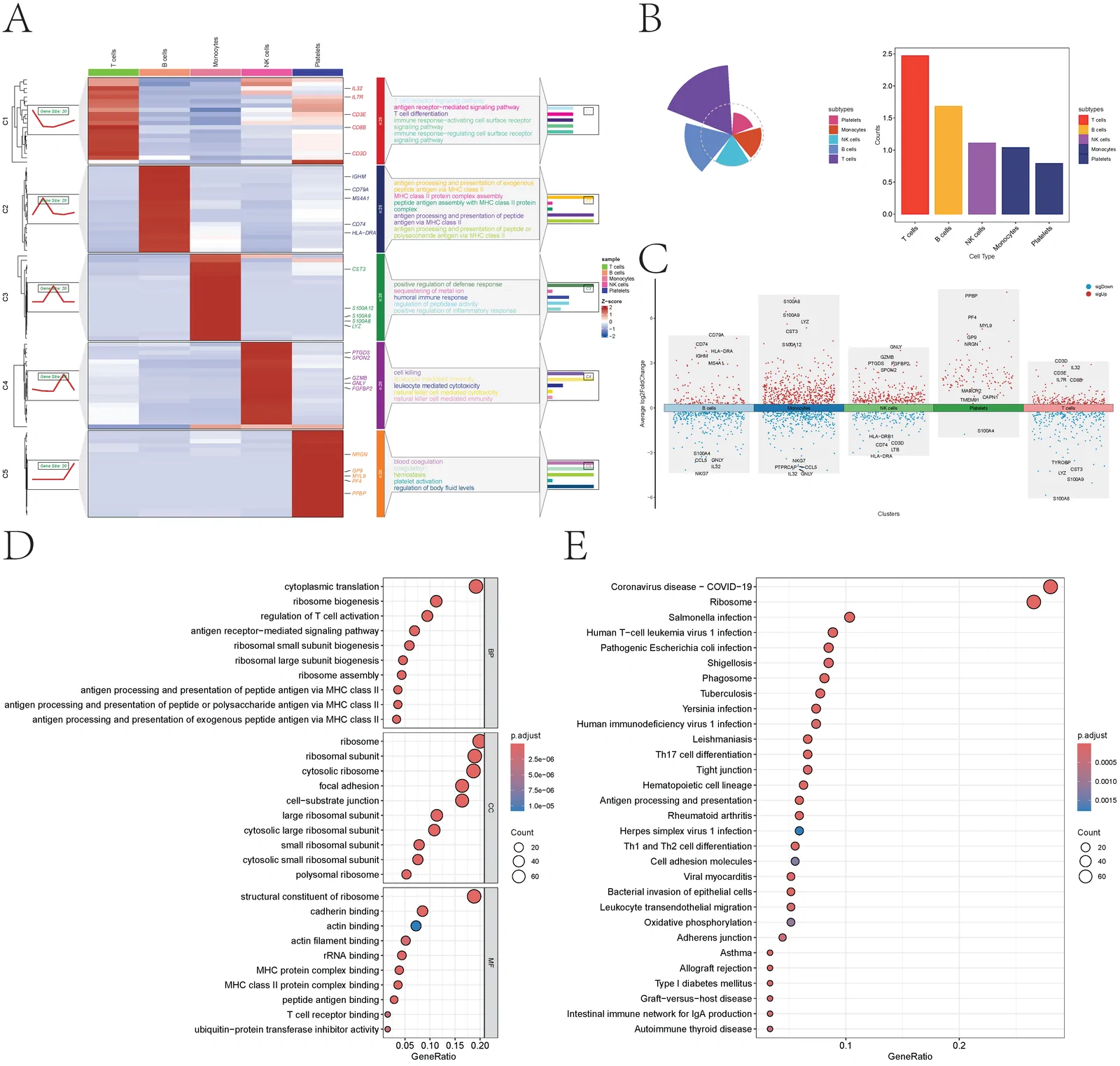
** Differential enrichment and disease contribution of cell subsets. (A)** Differential enrichment of five cell types. **(B)** Disease contribution scores of the five cell types, with T cells showing the highest score. **(C)** Differentially expressed genes across the five cell types; blue and red indicate downregulated and upregulated genes, respectively. **(D-E)** GO and KEGG enrichment analyses performed with clusterProfiler.

We then analyzed 2000 highly variable genes and identified the 10 genes with the highest standard deviations (Figure S1C). The data were standardized, homogenized, subjected to PCA and analysis via Harmony and processed successively (Figure S1D-F). A total of 7 subgroups were obtained by using UMAP for dimensionality reduction (Figure 1A). In this study, each subtype was further annotated, and 7 subgroups were annotated into 5 cell categories: T cells, NK cells, B cells, monocytes, and platelets (Figure 1B). A dot plot of canonical markers for the five cell types is shown in Figure 1C, and the corresponding cell-type proportions are shown in Figure 1D. Subpopulation differences and enrichment analysis were then conducted, and the enrichment heatmap and enrichment annotation of the average expression of the cell subpopulations were displayed using ClusterGVis (Figure 2A). The results revealed that that the differentially expressed genes (DEGs) associated with T cells were associated with the T-cell receptor signaling pathway, T-cell differentiation, the immune response-activating cell surface receptor signaling pathway, the antigen receptor-mediated signaling pathway, the immune response-regulating cell surface receptor signaling pathway and other pathways.

### Determination of the contribution of cell subsets to disease

We evaluated the contribution of different cell subpopulations to disease by considering changes in cell numbers and gene expression. The control group with fewer than 3 cells was not included in the analysis. First, we selected significantly upregulated DEGs between the disease group and the control group to describe changes in this process. We then determined the FCscore, which measures changes in the number and expression levels of characteristic genes during biological processes. T cells had the highest disease contribution score (Figure 2B).

### T-cell DEG identification and functional annotation

**Figure 3:**
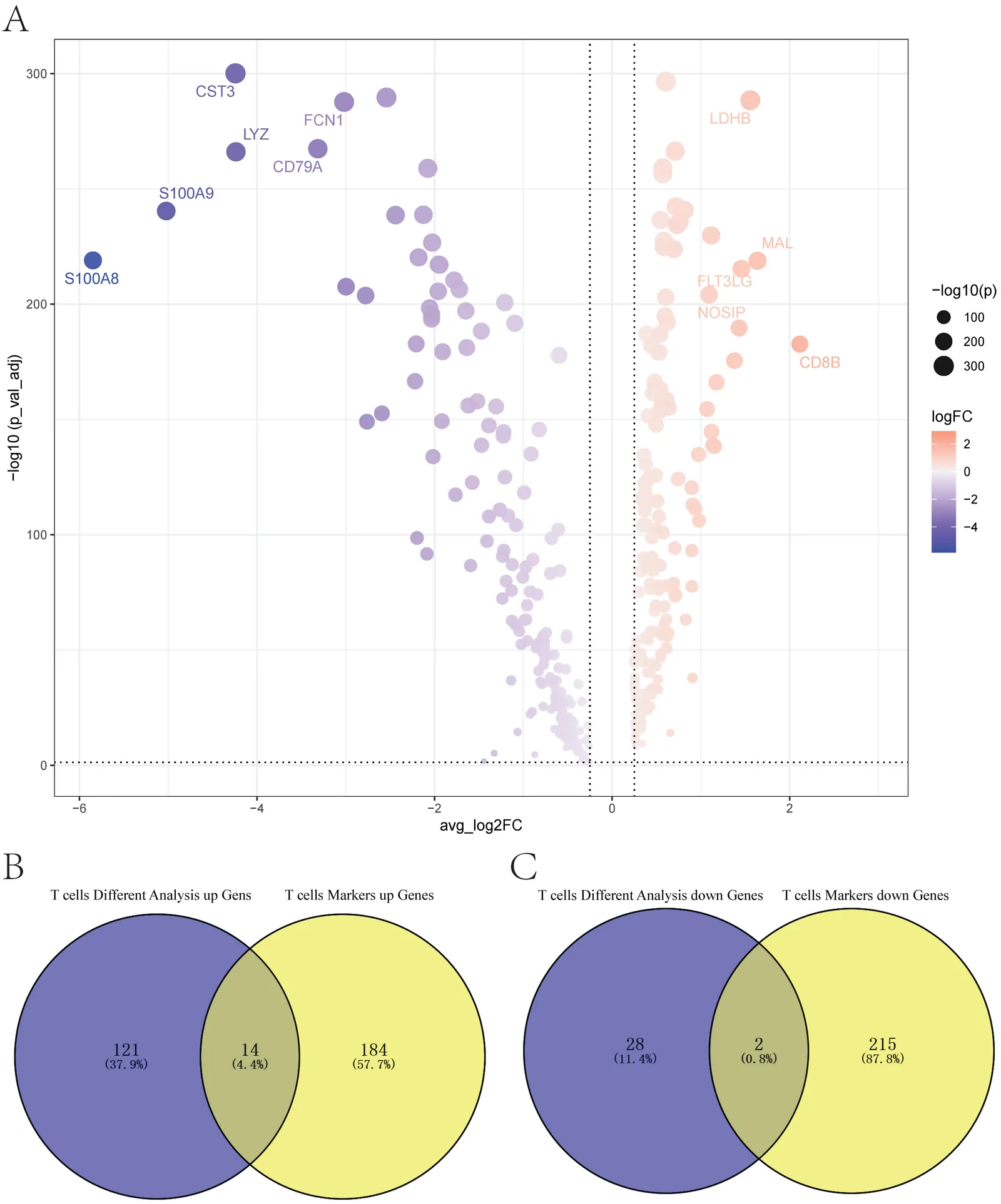
** Differential analysis of T cells. (A)** Volcano plot of T-cell marker genes; blue and red indicate downregulated and upregulated genes, respectively. **(B)** Intersection of genes upregulated in CAP T cells and upregulated T-cell marker genes. **(C)** Intersection of genes downregulated in CAP T cells and downregulated T-cell marker genes.

**Figure 4:**
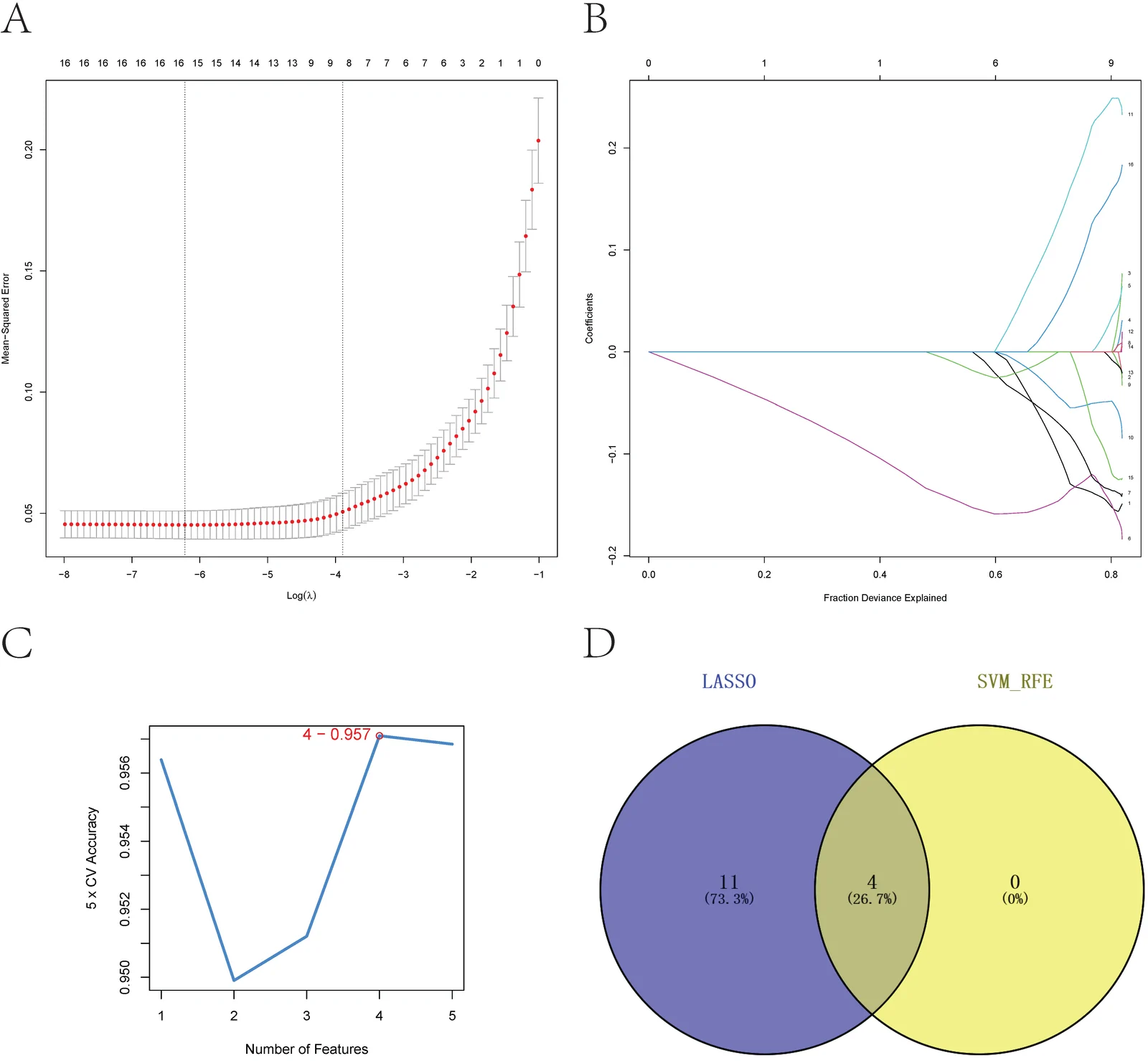
** LASSO and SVM-RFE feature selection. (A)** LASSO coefficient profiles. **(B)** Ten-fold cross-validation used to select lambda.min. **(C)** Four genes with the highest SVM-RFE accuracy. **(D)** Intersection of genes selected by LASSO and SVM-RFE.

Differential analysis of cell types was performed using the FindAllMarkers function (|log2FC| > 0.25 and adjusted P < 0.05) (Figure 3A-B). The ranked FindAllMarkers output for the T-cell cluster included FCER1G, SAT1, FTL, TYMP, TYROBP, CFD, CSTA, ALOX5AP, CTSD, and FCN1. Because FindAll-Markers returns both up- and downregulated genes, myeloid-associated genes such as TYROBP, FCN1, LYZ, S100A8, and S100A9 in this output were downregulated in the T-cell cluster and were not used as positive annotation markers. T-cell identity was supported by CD3D, CD3E, CD3G, and CD8B expression shown in Figure 1C and was cross-checked against the deposited cell metadata. DoubletFinder was used to remove predicted doublets; nevertheless, residual ambient RNA cannot be fully excluded. We screened the top 200 marker genes for functional analysis. GO enrichment analysis revealed significant enrichment in biological processes such as antigen processing and presentation, leukocyte cell-cell adhesion, and positive regulation of T-cell activation; cellular components such as vacuolar lumen and secretory granule membrane; and molecular functions such as protein phosphatase binding and cytokine receptor binding (Figure 4A). Kyoto Encyclopedia of Genes and Genomes analysis revealed enrichment in pathways such as lysosome, phagosome, apoptosis, and phosphatidylinositol signaling (Figure 4B).

### Feature selection process of the LASSO regression and SVM algorithms

First, marker genes in different cell types were reviewed, and a marker gene volcano map of T cells was drawn (Figure 3A). Then, an exploratory cell-level differential-expression analysis was performed between CAP and control T cells, and the resulting upregulated and downregulated genes were compared with those identified in the marker-gene analysis. Because donor identity was not incorporated into the differential-expression model, these findings may be affected by pseudoreplication and should not be interpreted as confirmatory donor-level evidence. Fourteen shared upregulated genes and 2 shared downregulated genes were obtained. These 16 genes were used as candidate genes for subsequent analysis (Figure 3B-C). Next, we used LASSO regression and the SVM algorithm to screen out the candidate genes associated with CAP. The LASSO regression results revealed 15 genes that were associated with CAP. In addition, we screened 4 candidate genes associated with CAP by SVM (Figure 4A-C). The candidate genes selected by LASSO were compared to those identified using SVM, and four shared candidate biomarkers were identified (Figure 4D): CD8B, GZMM, MYL12B and TUBA1A. These four genes were defined as candidate biomarkers for subsequent analysis.

### Immune infiltration analysis

**Figure 5:**
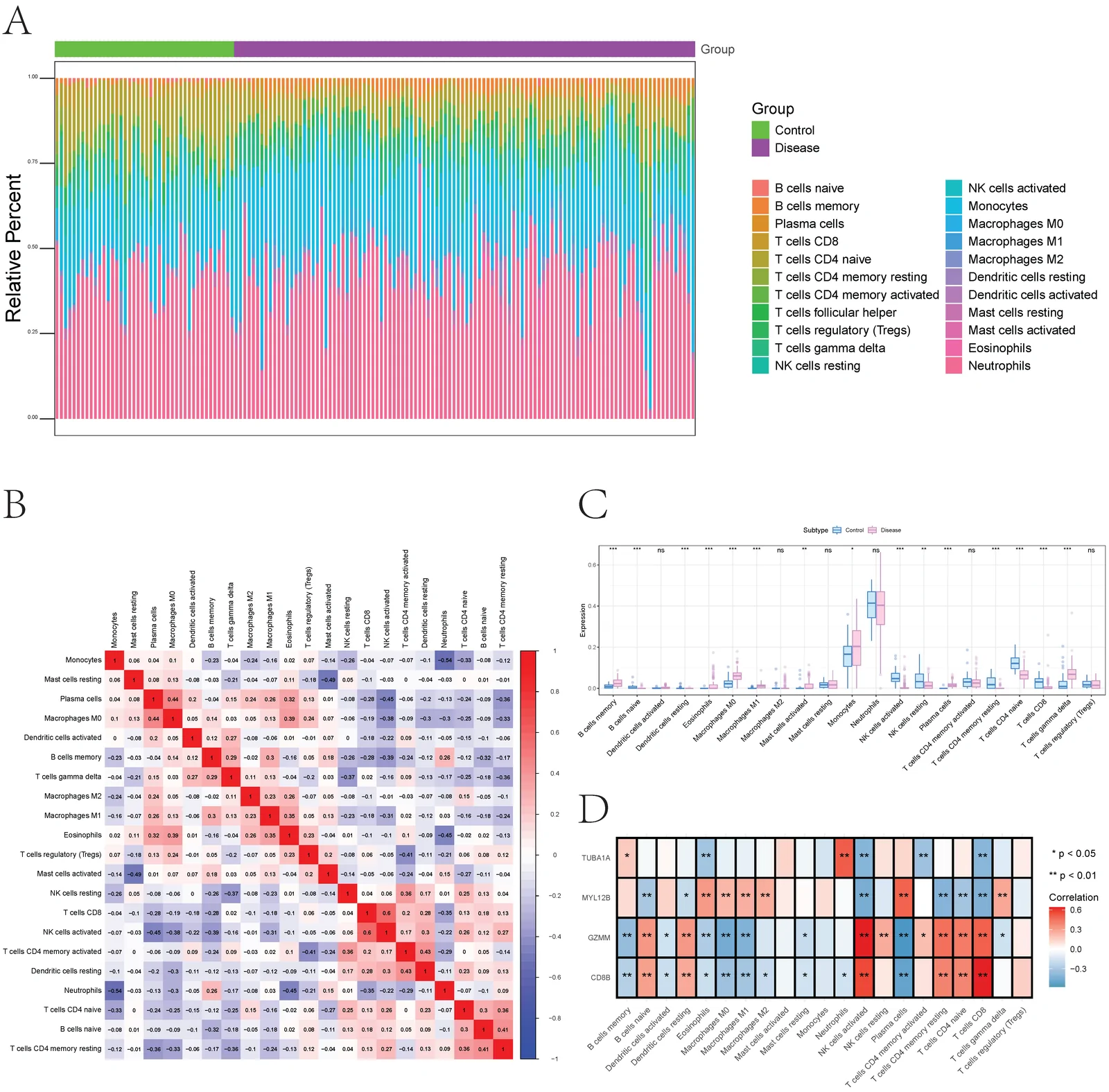
** Immune infiltration analysis. (A)** Relative proportions of immune-cell subsets. **(B)** Correlations among immune-cell fractions; blue and red indicate negative and positive correlations, respectively. **(C)** Differences in inferred immune-cell fractions between control and CAP samples. **(D)** Correlations between candidate biomarker expression and immune-cell fractions.

**Figure 6:**
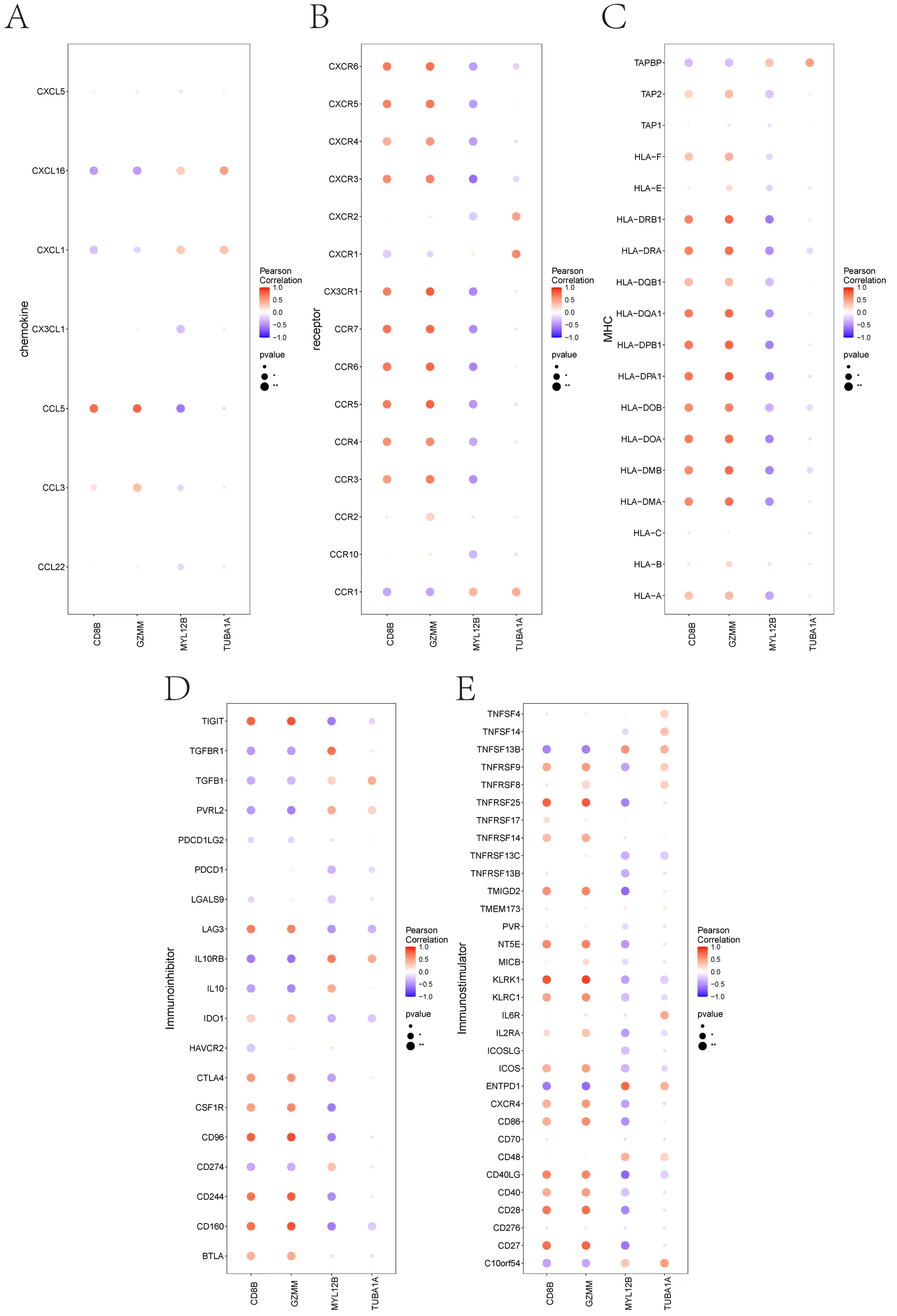
** Associations between candidate biomarkers and immune factors. (A-E)** Correlations of candidate biomarkers with chemokines, immunoinhibitors, immunostimulators, MHC molecules, and receptors.

The peripheral immune compartment comprises diverse leukocyte populations whose relative abundance may reflect host-response heterogeneity in CAP. We therefore used CIBERSORT to infer immune-cell distributions from the bulk transcriptomic dataset and explored correlations between estimated immune-cell fractions and candidate biomarkers (Figure 5A-B). Compared with controls, CAP samples showed increased eosinophils, activated mast cells, monocytes, and plasma cells, whereas resting CD4+ memory T cells and natural killer (NK) cells were reduced (Figure 5C). CD8B expression was positively correlated with naive B cells, CD8+ T cells, naive CD4+ T cells, resting CD4+ memory T cells, activated NK cells, and resting dendritic cells, but negatively correlated with memory B cells, plasma cells, macrophages, activated dendritic cells, resting mast cells, eosinophils, and neutrophils. GZMM showed positive correlations with naive B cells, CD8+ T cells, naive CD4+ T cells, resting/activated CD4+ memory T cells, NK cells, and resting dendritic cells, and negative correlations with memory B cells, plasma cells, gamma delta T cells, macrophages, activated dendritic cells, and mast cells. MYL12B was positively correlated with plasma cells, gamma delta T cells, macrophages, and eosinophils but negatively correlated with naive B cells, CD8+ T cells, naive CD4+ T cells, resting CD4+ memory T cells, activated NK cells, and resting dendritic cells. TUBA1A was positively correlated with memory B cells and neutrophils and negatively correlated with CD8+ T cells, activated CD4+ memory T cells, activated NK cells, and eosinophils (Figure 5D). These findings suggest that the candidate biomarkers are closely related to peripheral immune-cell composition in CAP (Figure 6A-E).

### GSEA

**Figure 7:**
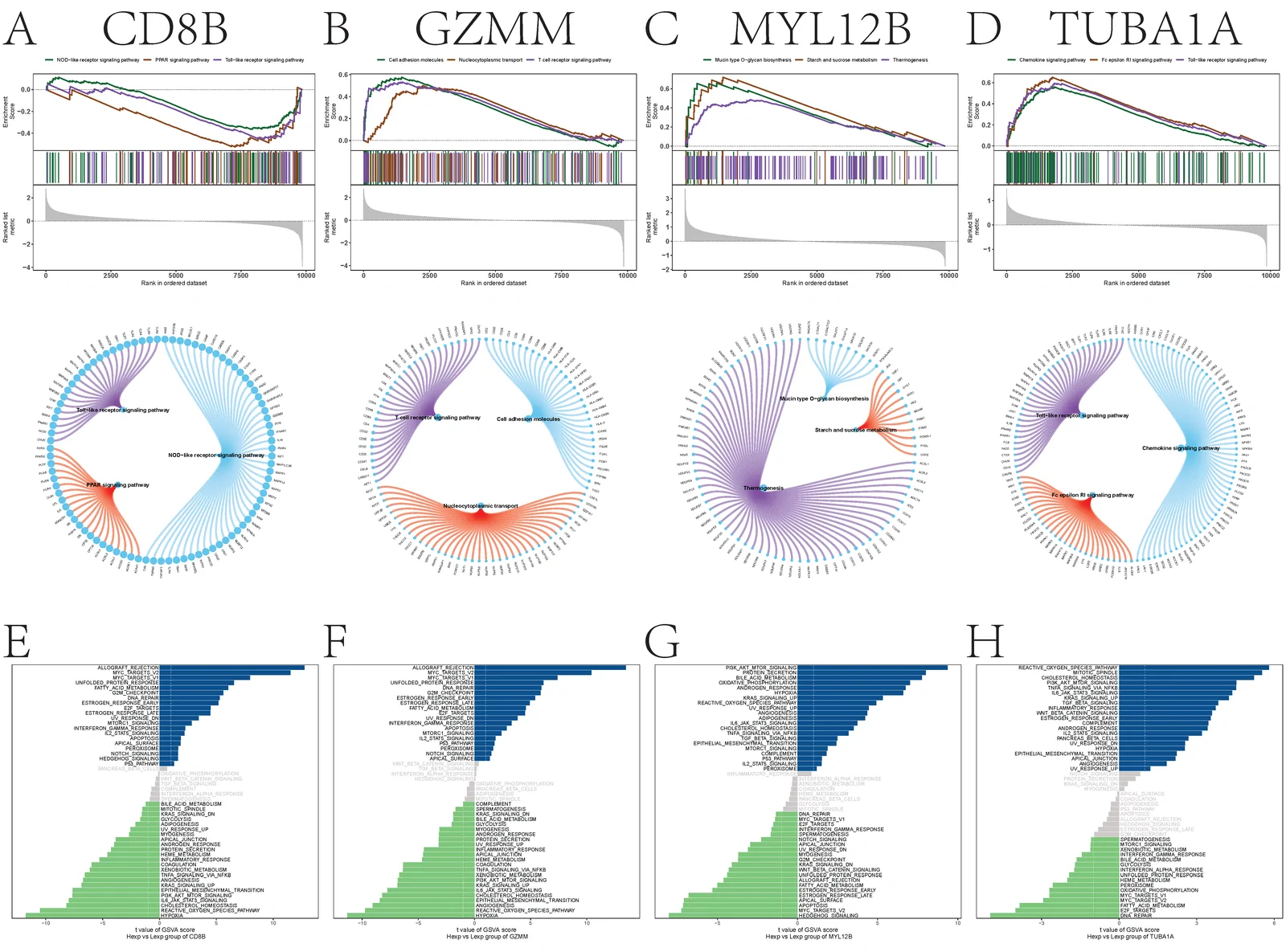
** GSEA and GSVA of candidate biomarkers. (A-D)** KEGG pathways associated with each candidate biomarker. **(E-H)** GSVA pathway signatures stratified by candidate-biomarker expression; blue and green indicate high- and low-expression groups, respectively. Hallmark gene sets were used as the reference.

Next, we examined signaling-pathway associations of the candidate biomarkers. The GSEA results revealed that CD8B was associated with the PPAR signaling pathway, Toll-like receptor signaling pathway, NOD-like receptor signaling pathway and other signaling pathways (Figure 7A). GZMM was associated with cell adhesion molecules, the T-cell receptor signaling pathway, the nucleocytoplasmic transport pathway and other signaling pathways (Figure 7B). MYL12B was associated with thermogenesis, starch and sucrose metabolism, mucin-type O-glycan biosynthesis and other signaling pathways (Figure 7C). TUBA1A was associated with the chemokine signaling pathway, Toll-like receptor signaling pathway, Fc epsilon RI signaling pathway and other signaling pathways (Figure 7D).

### GSVA

GSVA revealed that CD8B was associated with signaling pathways such as ALLOGRAFT_REJECTION and MYC_TARGETS_V2 (Figure 7E). GZMM was associated with signaling pathways such as ALLOGRAFT_REJECTION and MYC_TARGETS_V2 (Figure 7F). MYL12B was associated with signaling pathways, including PI3K_AKT_MTOR_SIGNALING and PROTEIN_SECRETION (Figure 7G). TUBA1A is enriched in signaling pathways such as REACTIVE_OXYGEN_SPECIES_PATHWAY and MITOTIC_SPINDLE (Figure 7H). These findings suggest that candidate biomarkers were associated with these pathway-level signatures.

### Relationships between candidate biomarkers and disease-related genes

**Figure 8:**
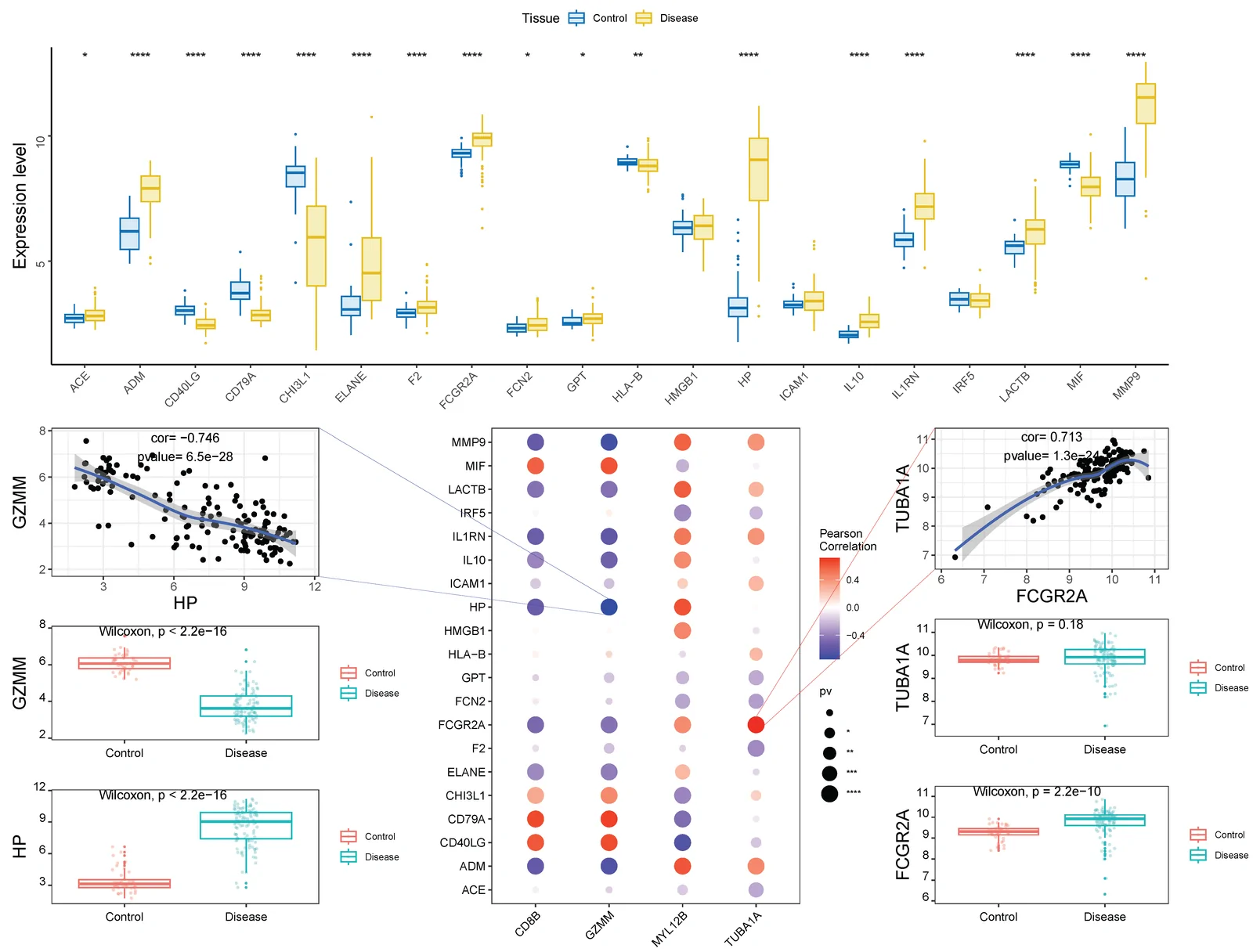
** Associations between candidate biomarkers and disease-related genes.** Top: Expression differences in disease-related genes; blue and yellow indicate control and CAP samples, respectively. Bottom: Pearson correlations between candidate biomarkers and disease-related genes; blue and red indicate negative and positive correlations, respectively.

We identified disease-related genes using the GeneCards database 26. Intergroup differences in disease-related gene expression were analyzed. In this study, 20 genes with high Relevance Score values and detectable transcriptomic expression were assessed. The expression levels of ACE, ADM, CD40LG, CD79A, CHI3L1, ELANE, F2, FCGR2A, FCN2, GPT, HLA-B, HP, IL10, IL1RN, LACTB, MIF, and MMP9 were significantly different between groups. Correlation analysis further revealed that the expression levels of the candidate biomarkers were significantly associated with disease-related genes; notably, TUBA1A and FCGR2A were positively correlated (r = 0.713), whereas GZMM and HP were negatively correlated (r = -0.746) (Figure 8).

### Coexpression patterns of candidate biomarkers in single cells and pseudotime analysis

**Figure 9:**
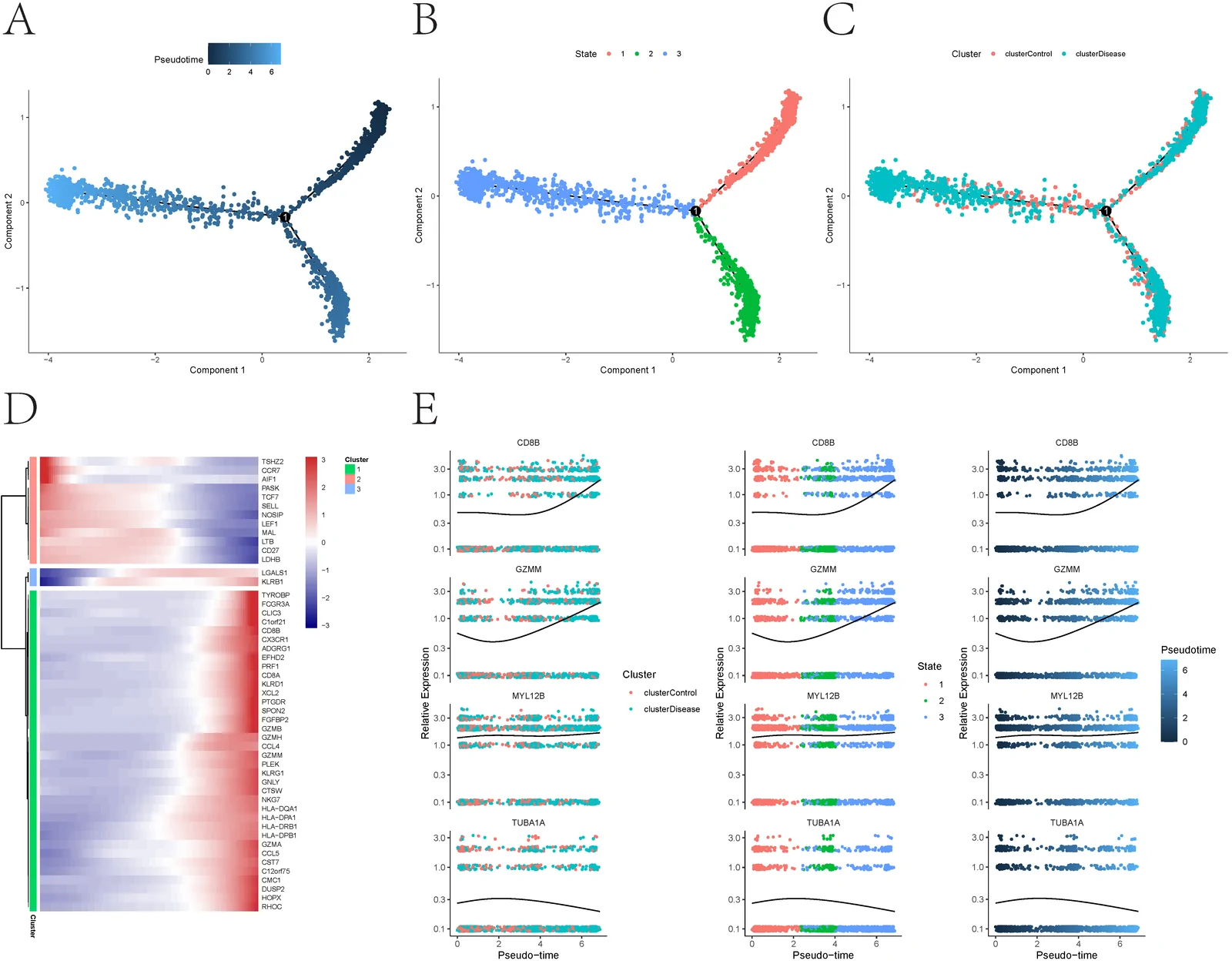
** Inferred T-cell trajectory. (A-C)** Pseudotime analysis and inferred trajectory of T cells. **(D)** Gene-expression dynamics across T-cell trajectory branches. **(E)** Relationship between candidate biomarker expression patterns and inferred pseudotime.

We selected the genes FCGR2A and HP, whose expression levels were significantly correlated with one another, for correlation analysis between candidate biomarkers and disease-related genes and explored the coexpression network of candidate biomarkers and disease-related genes through correlation analysis. A positive expression association between TUBA1A and FCGR2A was observed. A negative expression association between GZMM and HP was observed in the single-cell data (Figure S2-S5). Then, we conducted a pseudotime analysis of candidate biomarkers for T cells, first calculating the degree of similarity of the gene expression profiles among cells and constructing the cell differentiation trajectory. The inferred trajectory was visualized to compare transcriptional states and gene-expression patterns across pseudotime. Pseudotime values and states represent Monocle-derived transcriptional ordering and trajectory branches, respectively, rather than measured chronological time. Control cells were concentrated at lower pseudotime values, whereas CAP cells were concentrated at higher pseudotime values (Figure 9A-C). Through the calculation, we also selected and visualized the batch of genes that changed the most in terms of the pseudotime difference. The horizontal coordinate is the pseudotime value, and the vertical coordinate is the selected genes, which are divided into 3 clusters by default according to the changes in genes. We found that TSHZ2, CCR7, AIF1 and other genes were predominantly expressed at lower pseudotime values, whereas TYROBP, FCGR3A, CLIC3 and other genes were predominantly expressed at higher pseudotime values (Figure 9D). We subsequently demonstrated changes in the expression of candidate biomarkers across pseudotime. The results revealed that the expression of the CD8B and GZMM genes first decreased but then gradually increased with increasing pseudotime. The expression of the MYL12B gene was relatively stable across pseudotime. The expression of the TUBA1A gene first increased but then decreased with increasing pseudotime (Figure 9E).

### Evaluation of candidate biomarkers in an independent clinical cohort

To evaluate PBMC-level expression of the selected candidate biomarkers, we performed quantitative real-time PCR (qRT-PCR) assays on peripheral blood mononuclear cells (PBMCs) isolated from an independent validation cohort comprising 36 CAP patients and 18 healthy controls.

**Figure 10:**
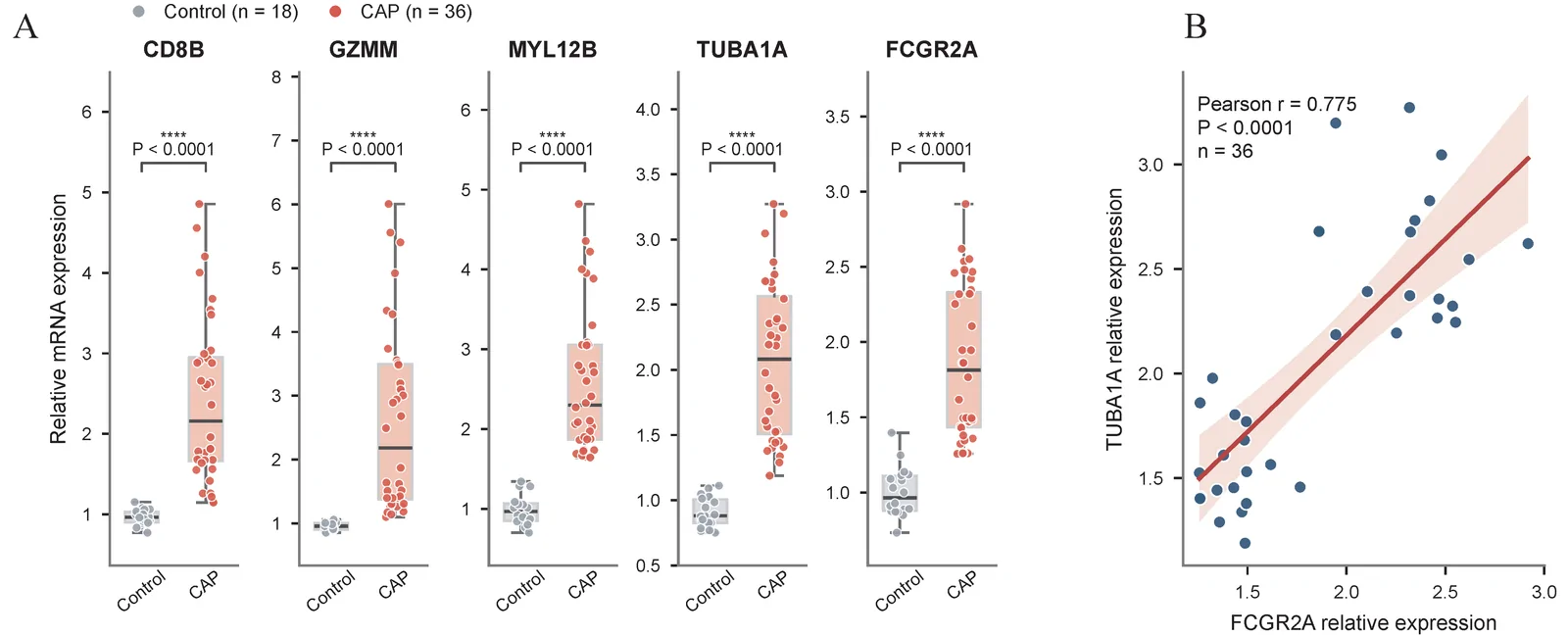
** Experimental validation of selected genes in an independent clinical cohort. (A)** The relative mRNA expression levels of four candidate biomarkers (CD8B, GZMM, MYL12B, and TUBA1A) together with one CAP-associated gene highlighted by network analysis (FCGR2A) were quantified by qRT-PCR in peripheral blood mononuclear cells (PBMCs) from CAP patients (n = 36) and healthy controls (n = 18). Data are presented as box plots overlaid with individual data points. The central line represents the median, the box bounds represent the interquartile range (IQR), and the whiskers extend to 1.5 times the IQR. Statistical significance was determined using a two-sided Welch's t-test. ****P < 0.0001.** (B)** Scatter plot showing the positive Pearson correlation between TUBA1A and FCGR2A mRNA expression in the CAP cohort. The line and shaded band denote the linear fit and 95% confidence interval. The reported coefficient is the Pearson correlation coefficient.

Consistent with the in silico analyses, qRT-PCR demonstrated significant upregulation of the four candidate biomarkers identified by machine learning. As illustrated in Figure 10A, CD8B, GZMM, MYL12B, and TUBA1A were all significantly elevated in the CAP group compared with healthy controls (P < 0.0001). Because network analysis also highlighted FCGR2A as a CAP-associated gene, this transcript was additionally measured and was likewise increased in CAP PBMCs.

In addition to expression profiling, we examined co-expression patterns of selected gene pairs to assess expression associations predicted by the network analysis. As shown in Figure 10B, a strong positive Pearson correlation was observed between TUBA1A and FCGR2A expression (r = 0.775, P < 0.0001). This experimental finding aligns with our computational results, supporting an association between cytoskeletal and Fc receptor-related expression programs and generating a hypothesis for future mechanistic testing.

## Discussion

This study systematically reveals peripheral T-cell immune dysregulation in CAP using integrated single-cell and bulk transcriptomic analysis. We identified four T-cell-associated candidate blood biomarkers (CD8B, GZMM, MYL12B, TUBA1A), whose differential expression was further evaluated by qRT-PCR in an independent clinical cohort. Previous transcriptomic studies of CAP mainly focused on innate immunity or single-omics analysis. Our study is the first to combine scRNA-seq, bulk RNA-seq, machine learning, and clinical qRT-PCR to identify T-cell-related candidate biomarkers in CAP peripheral blood, providing a more comprehensive and reliable screening strategy. Single-cell analysis confirmed that T cells show the highest disease contribution score in CAP peripheral blood, suggesting a prominent association with altered adaptive immune states. T cells in CAP exhibited distinct inferred transcriptional states and were enriched in antigen processing and immune activation pathways, consistent with systemic immune imbalance.

Despite advances in antimicrobial therapy and supportive care, CAP remains a formidable public health challenge characterized by persistently high morbidity and mortality rates 2,10. Severe CAP is a common disease in intensive care units (ICUs), accounting for 17-21% of hospitalized CAP patients, and the 30-day and 6-month case fatality rates of SCAP are as high as 27% and 39%, respectively 11. Despite significant advances in molecular diagnostics, the management of CAP continues to face challenges owing to our limited understanding of the intricate pathogen‒host interaction networks. In this study, by combining single-cell sequencing and bulk transcriptomic profiling, we identified candidate genes associated with CAP and generated hypotheses for further mechanistic investigation.

CAP is characterized by dynamic immune infiltration that critically influences disease progression and outcomes. Neutrophil infiltration and cytokine storms are hallmarks of acute CAP, and their overactivation contributes to tissue damage and ARDS 12. Conversely, T-cell exhaustion and regulatory T-cell expansion may suppress pathogen clearance, enabling bacterial persistence 13. Single-cell RNA sequencing data (GSE164948) revealed that T cells constitute the most significantly altered immune cell population in CAP. Functional enrichment analysis of 415 T-cell-specific marker genes revealed key immune pathways, including antigen receptor signaling, T-cell activation, and cell adhesion dynamics. KEGG pathway analysis further highlighted the involvement of oxidative phosphorylation and rheumatoid arthritis-associated immunity, suggesting that T-cell metabolic reprogramming and chronic immune activation are involved in CAP pathogenesis. The increased percentages of eosinophils, activated mast cells, and plasma cells in CAP patients suggest hyperactive humoral and allergic-like responses, whereas the reduced percentages of NK cells and CD4+ memory T cells indicate impaired cytotoxic and adaptive immune surveillance 14,15. These findings align with the clinical observation of severe inflammatory responses in CAP, potentially driven by dysregulated immune cell interactions.

We identified CD8B, GZMM, MYL12B, and TUBA1A as the genes most significantly dysregulated in CAP. These genes exhibited distinct immune cell interaction profiles, showing associations with the immune microenvironment. Notably, our data revealed that CD8B and GZMM expression was positively correlated with the percentages of effector T/NK cells but negatively correlated with that of immunosuppressive macrophages (M2), indicating associations with both effector-cell and macrophage fractions 16. The positive correlation of MYL12B expression with the percentages of classically activated M1 macrophages and eosinophil populations, combined with its established role in actin polymerization dynamics, is consistent with potential involvement in inflammatory cell chemotaxis and activation 17,18. However, its inverse association with cytotoxic T lymphocyte (CTL) and NK cell percentages also indicates an inverse association with cytotoxic-cell fractions. TUBA1A expression was correlated with the percentages of neutrophils and CD8+ memory T cells, and its involvement in cytoskeletal dynamics was associated with neutrophil-related changes during CAP 19. Notably, our wet-lab validation demonstrated a high degree of consistency with the in silico predictions. All five assayed genes showed significant upregulation in clinical samples, supporting consistency between the computational and PBMC-level findings. These findings highlight further evaluation of these genes as candidate blood biomarkers in CAP.

Transcriptomic studies of CAP have proven valuable in elucidating its pathogenic mechanism. For example, Song et al. identified transcriptomic biomarkers, including otoferlin (OTOF), MS4A4A, and SIGLEC1, as potential prognostic indicators for severe CAP 20. Hao et al. utilized transcriptomic data and reported that PANoptosis feature genes demonstrated high diagnostic accuracy for CAP 21. In the present study, we analyzed disease-associated genes from the GeneCards database and identified 20 disease-related genes with significant expression differences between CAP patients and controls, including FCGR2A and HP. TUBA1A expression showed a strong positive correlation with that of FCGR2A, indicating a positive expression association during the host response. Conversely, the negative correlation between GZMM and HP expression represents an inverse expression association and should be considered a hypothesis rather than a mechanistic conclusion. Single-cell analysis suggested a negative association between GZMM and HP within specific cell subsets. This computational association was not evaluated in the revised qRT-PCR validation panel.

Furthermore, we performed pseudotime trajectory analysis to reveal distinct immune cell differentiation patterns. The results revealed that control cells were enriched at lower inferred pseudotime values, whereas CAP cells were enriched at higher inferred pseudotime values, consistent with distinct transcriptional states. The dynamic expression patterns of candidate biomarkers, including a decrease followed by the recovery of CD8B levels and stable high expression of MYL12B, were associated with inferred T-cell state changes 22,23. These cross-sectional associations do not establish temporal differentiation or CAP progression.

There are several limitations associated with this study. The CAP-versus-control differential-expression analysis within T cells treated individual cells rather than donors as the statistical units and did not account for donor identity. Because cells from the same donor are not statistically independent, the analysis may be affected by pseudoreplication and inflated statistical significance. These findings should therefore be interpreted as exploratory and require confirmation using donor-aware pseudobulk or mixed-effects analyses in larger independent single-cell cohorts. Besides, the secondary scRNA-seq analysis used GEO-deposited processed matrices rather than an original Seurat object or author analysis code, which limits exact reconstruction of upstream preprocessing. The scRNA-seq dataset was also relatively small, and our clinical validation cohort, while supporting the bioinformatic findings at the PBMC mRNA level, was derived from a single center. Future multi-center prospective studies with larger populations are warranted to further evaluate the reproducibility and diagnostic performance of these candidates. Second, the validation assessed differential mRNA expression in unsorted PBMCs and did not include ROC or calibration analyses. Accordingly, the results do not establish clinical diagnostic performance or a T-cell-specific cellular source, and differences in PBMC composition may partly account for the observed expression changes. Since mRNA abundance does not always linearly correlate with protein expression, and the specific molecular functions were not verified through *in vivo* function experiments, further investigations using protein-level assays and mechanistic studies are needed to fully elucidate the regulatory networks of these candidate biomarkers in the CAP immune microenvironment. In addition, the qRT-PCR CAP and control groups were not age matched, so age-related transcriptional differences may have confounded the observed group differences.

## Conclusions

In conclusion, our study identifies four T-cell-associated candidate blood biomarkers for CAP. These findings improve understanding of CAP immune pathogenesis and warrant multicenter diagnostic evaluation and mechanistic investigation.

## Supplementary Material

Supplementary figures and tables.

## Figures and Tables

**Table 1:** Demographic characteristics of the independent qRT-PCR validation cohort

Characteristic	CAP (n = 36)	Healthy controls(n = 18)	P value
Age, years, median (IQR)	72.5 (61.0-81.0)	37.0 (33.0-49.0)	<0.001
Male sex, n (%)	20 (55.6)	9 (50.0)	0.776

Data are median (interquartile range) or n (%). Age was compared using the two-sided Mann-Whitney U test and sex using Fisher's exact test. Participants were not individually matched.

## Data Availability

The datasets analysed during the current study are available in the NCBI Gene Expression Omnibus repository under accession numbers GSE65682 and GSE164948 [24,25]. The processed count matrices, sample annotation, and cell-level cluster metadata used for the GSE164948 secondary analysis are available as GEO supplementary files. The clinical qRT-PCR data generated during the current study are not publicly available due to potentially identifiable clinical information but are available from the corresponding author on reasonable request and with approval of the relevant ethics committee.

## Abbreviations

CAP: community-acquired pneumonia
PBMC: peripheral blood mononuclear cell
scRNA-seq: single-cell RNA sequencing
GEO: Gene Expression Omnibus
DEG: differentially expressed gene
LASSO: least absolute shrinkage and selection operator
SVM-RFE: support vector machine-recursive feature elimination
CIBERSORT: Cell-type Identification By Estimating Relative Subsets Of RNA Transcripts
GSEA: gene set enrichment analysis
GSVA: gene set variation analysis
UMAP: uniform manifold approximation and projection
PCA: principal component analysis
qRT-PCR: quantitative real-time polymerase chain reaction
