# Supplementary material for: Integrative single-cell and bulk transcriptomic profiling identify peripheral T-cell-associated candidate biomarkers in community-acquired pneumonia
Source: Int J Med Sci. 2026 Aug 11;23(9):3010–26. doi: 10.7150/ijms.136006 (PMC13539919; doi:10.7150/ijms.136006)
Supplement: Supplementary file 1 [file ijmsv23p3010s1.pdf]

Integrative single-cell and bulk transcriptomic profiling identifies peripheral T-cell-associated candidate biomarkers in community-acquired pneumonia

Supplementary Materials

Contents

Supplementary Table S1. Primer sequences used for qRT-PCR.
Supplementary Figures 1-5.

Supplementary Table S1. Primer sequences used for qRT-PCR.

Gene Symbol	Forward Primer (5' -> 3')	Reverse Primer (5' -> 3')
CD8B	CAAGCCGGTTCATTCTCAATCTC	GAAGGAAATCAACCACACTCAGC
GZMM	GGTCTCACCTTCCACATCAAGG	ACTTTCCCGTCCAGCTGAAG
MYL12B	CTGAAGATGTCATCAGAAACGCC	CCACTTCCTCATCTGTAAACCGA
TUBA1A	TGGAGGAAGGTGAGTTTTCAGAG	CCTCTCCTTCAACAGAATCCACA
FCGR2A	GGTCAAGGTCACATTCTTCCAGA	GTTTCCTGTGCAGTGGTAATCAC
HP	CACCCTAACTACTCCCAGGTAGA	ATCCTTTGAAGGTAGGCAGATGG
GAPDH	AAGCTCATTTCCTGGTATGACAA	CTTACTCCTTGGAGGCCATGT

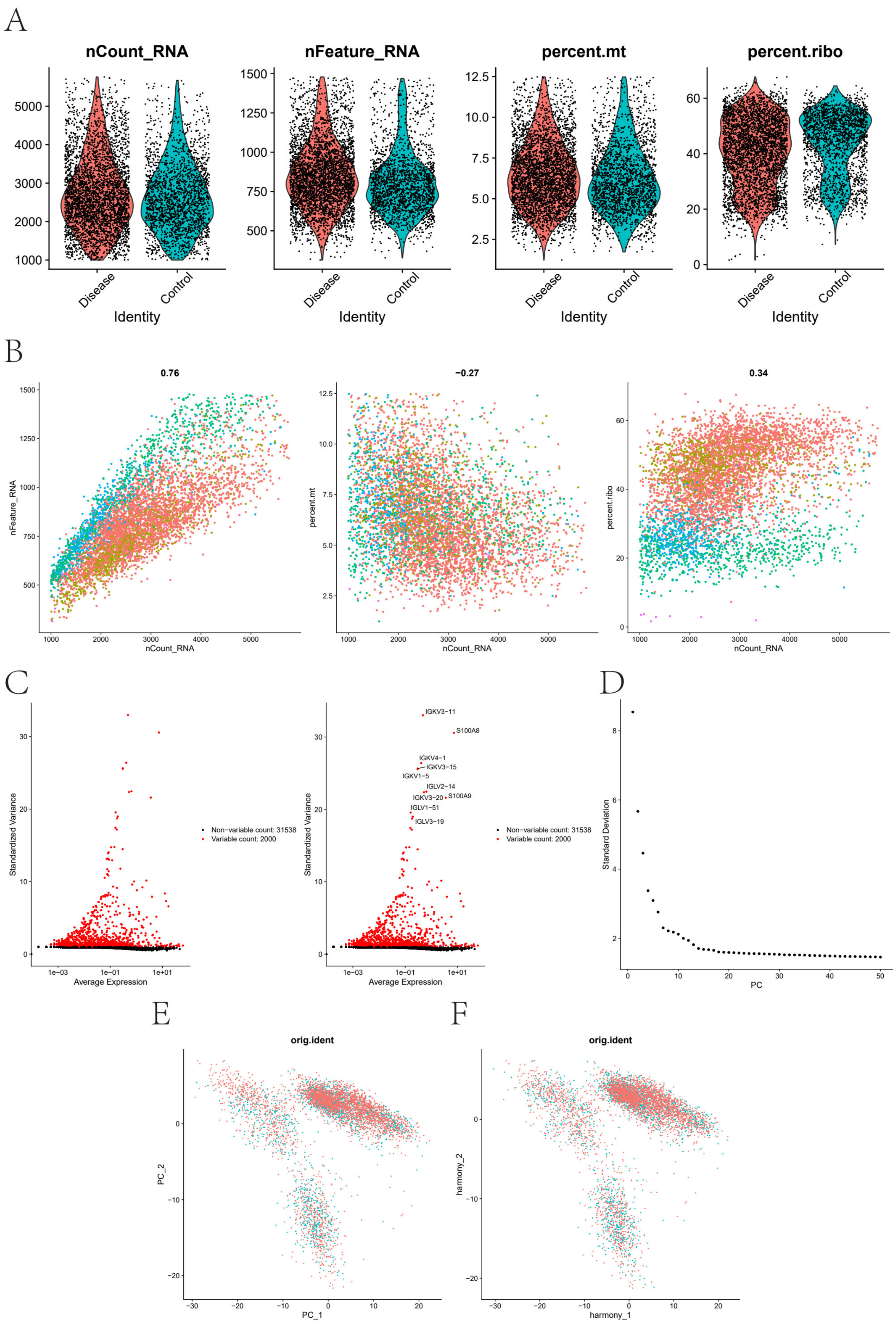

Supplementary Fig. 1 Single-cell preprocessing. (A) Distributions of nCount_RNA, nFeature_RNA, percent.mt, and percent.ribo across groups. (B) Pairwise relationships among sequencing depth, detected genes, mitochondrial percentage, and ribosomal percentage. (C) Highly variable genes. (D) Principal-component variance. (E-F) PCA before and after Harmony integration, colored by sample.

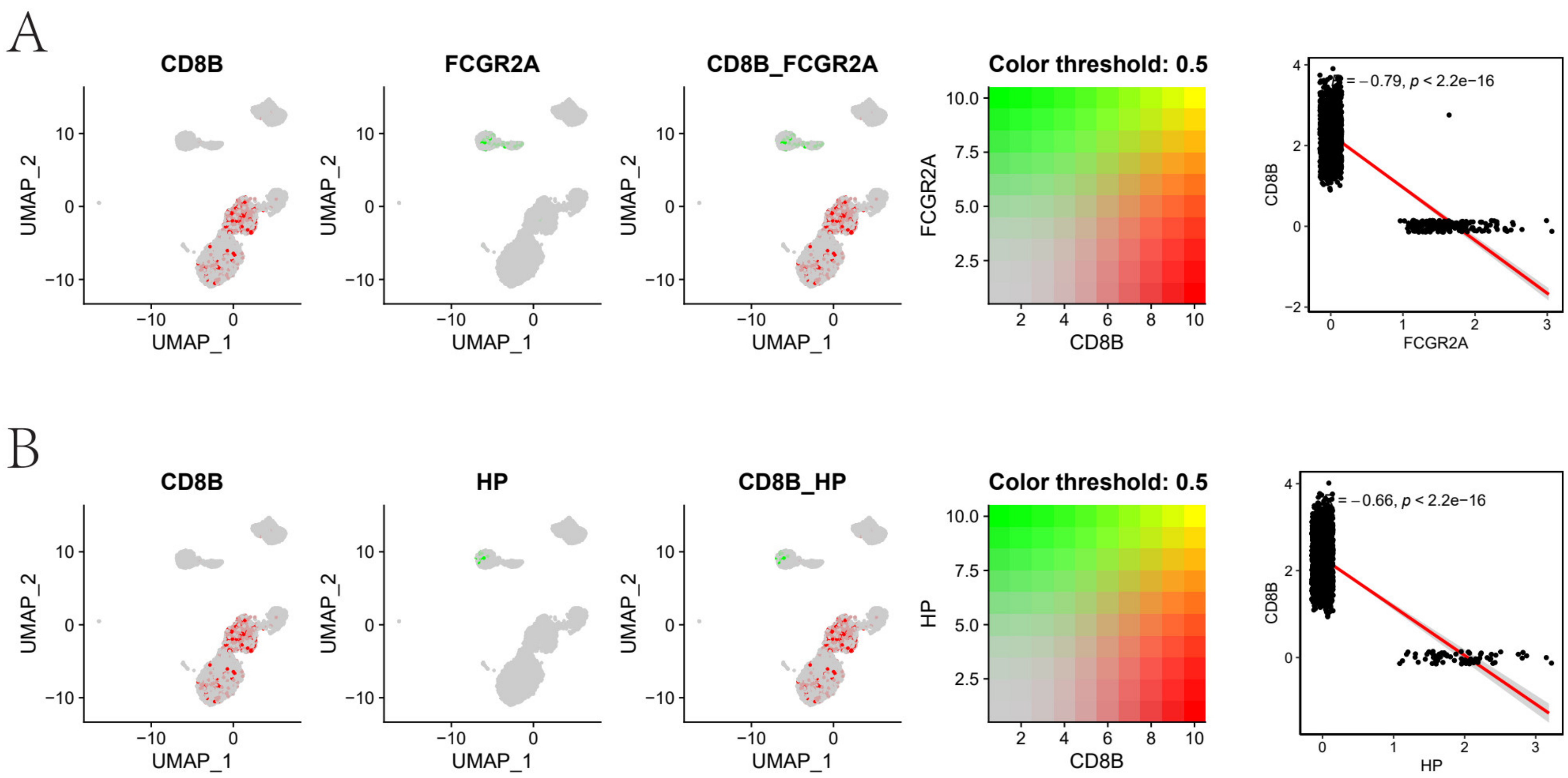

Supplementary Fig. 2 Single-cell co-expression analysis of CD8B with FCGR2A and HP. (A) Single-cell expression and co-expression patterns with FCGR2A. (B) Single-cell expression and co-expression patterns with HP; scatter plots show the corresponding expression correlations.

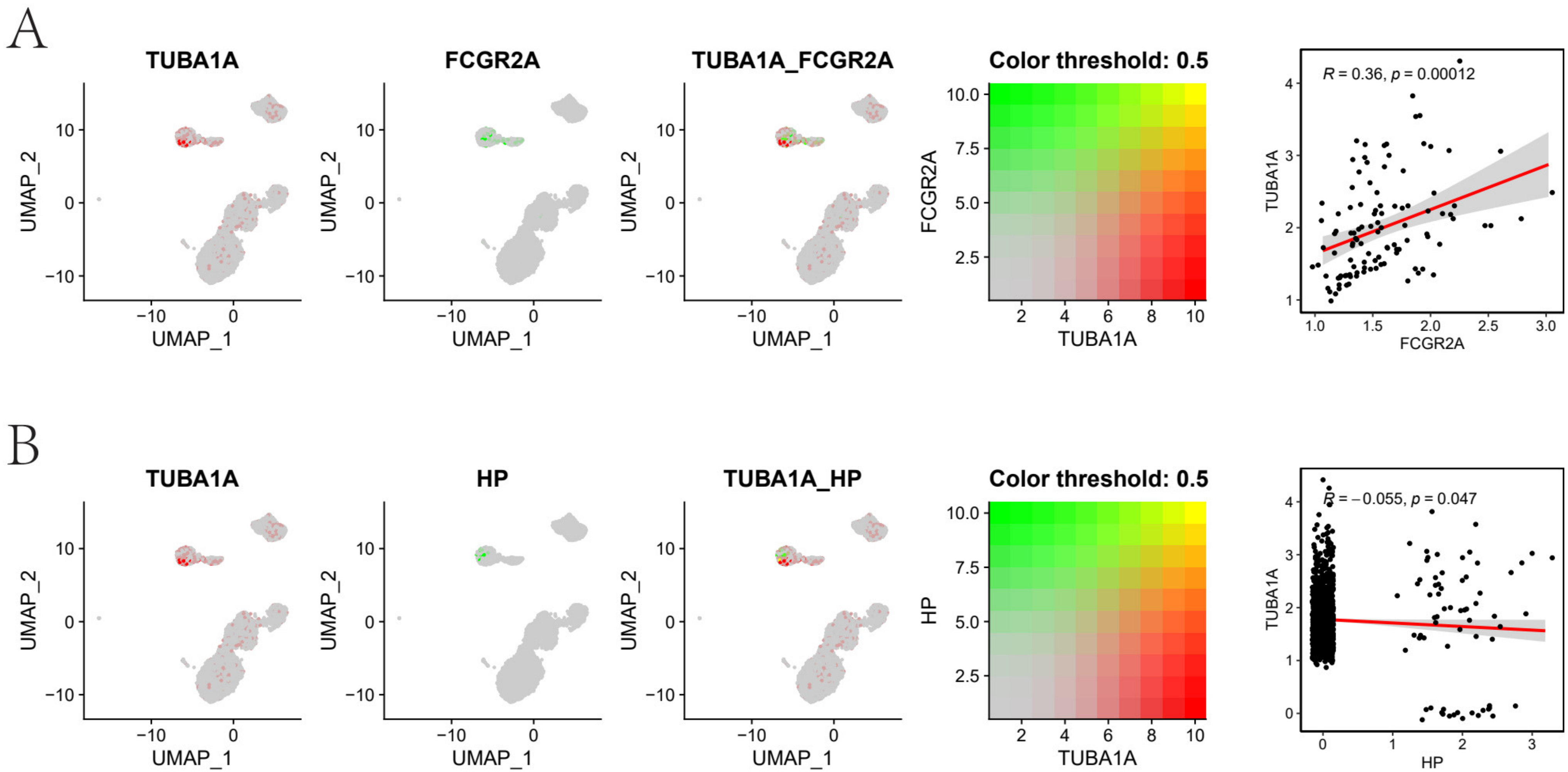

Supplementary Fig. 3 Single-cell co-expression analysis of TUBA1A with FCGR2A and HP. (A) Single-cell expression and co-expression patterns with FCGR2A. (B) Single-cell expression and co-expression patterns with HP; scatter plots show the corresponding expression correlations.

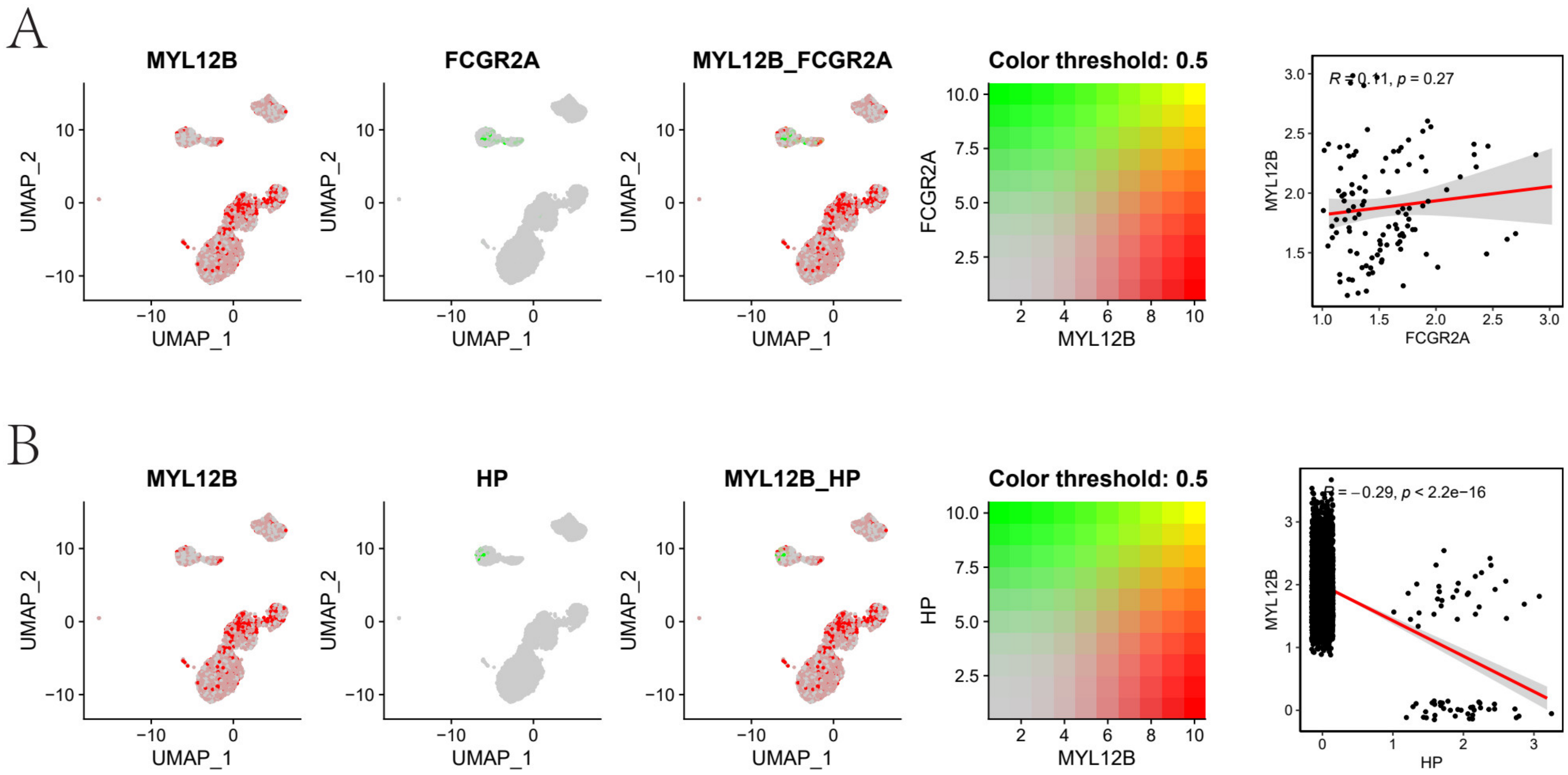

Supplementary Fig. 4 Single-cell co-expression analysis of MYL12B with FCGR2A and HP. (A) Single-cell expression and co-expression patterns with FCGR2A. (B) Single-cell expression and co-expression patterns with HP; scatter plots show the corresponding expression correlations.

A

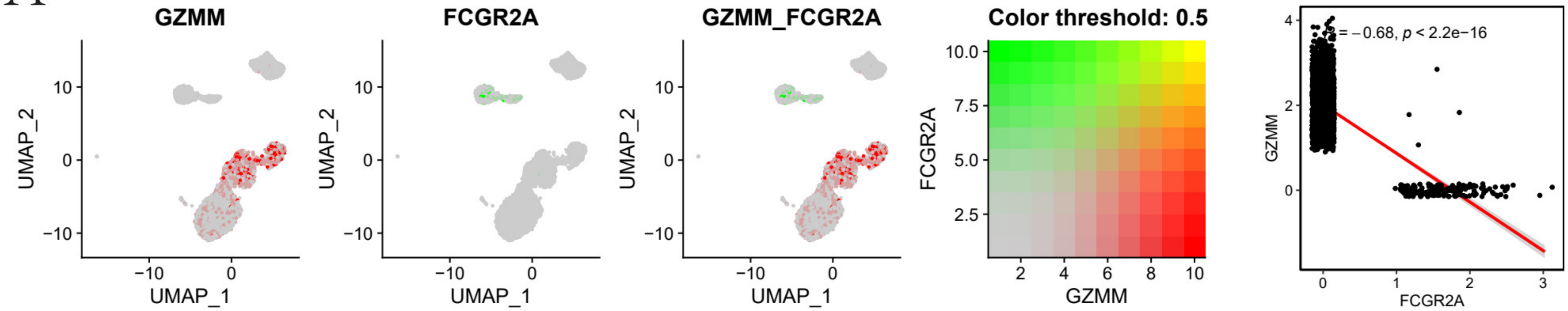

B

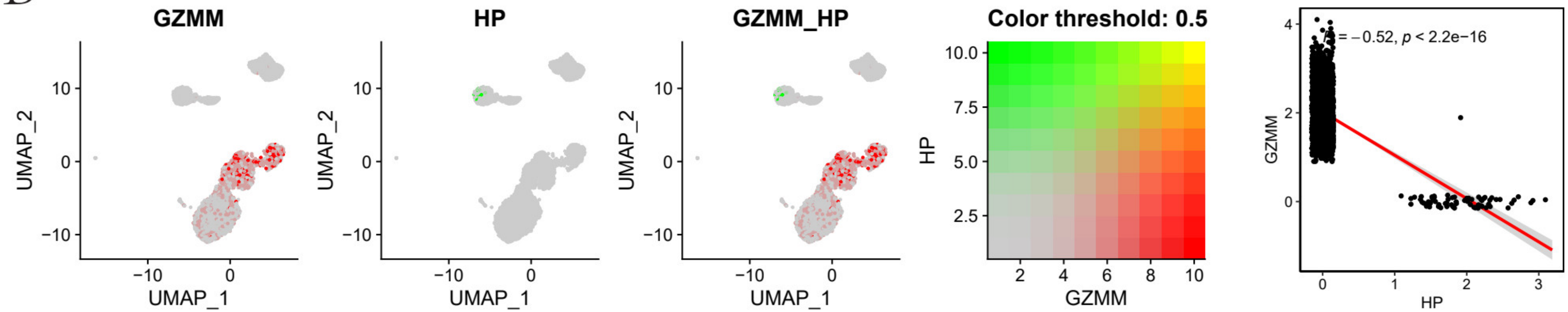

Supplementary Fig. 5 Single-cell co-expression analysis of GZMM with FCGR2A and HP. (A) Single-cell expression and co-expression patterns with FCGR2A. (B) Single-cell expression and co-expression patterns with HP; scatter plots show the corresponding expression correlations.
